# Clinical Efficacy of Topical Ziyun Ointment in Improving Skin Hydration and Reducing Transepidermal Water Loss in Psoriasis

**DOI:** 10.7150/ijms.118035

**Published:** 2025-09-12

**Authors:** Yu-Ping Hsiao, Wen-Li Ou, Chun-Te Lu, Jiunn-Liang Ko, Chih-Ting Hsu, Sheau-Chung Tang

**Affiliations:** 1 Institute of Medicine, School of Medicine, Chung Shan Medical University, Taichung, 402, Taiwan.; 2 Department of Dermatology, Chung Shan Medical University Hospital, Taichung, 402, Taiwan.; 3 School of Medicine, Chung Shan Medical University, Taichung, 402, Taiwan.; 4 Institute of Medicine, School of Medicine, College of Medicine, National Yang Ming Chiao Tung University, Taipei, 112, Taiwan.; 5 Division of Plastic and Reconstructive Surgery, Department of Surgery, Taichung Veterans General Hospital, Taichung, 407, Taiwan.; 6 Department of Medical Oncology and Chest Medicine, Chung Shan Medical University Hospital, Taichung 402, Taiwan.; 7 Manufacturing class, Puli Brewery, Taiwan Tobacco & Liquor Corporation, Nantou, 545, Taiwan.; 8 Department of Nursing, National Taichung University of Science and Technology, Taichung 406, Taiwan.

**Keywords:** psoriasis, Ziyun Oointment, erythema, PASI scores, Transepidermal Water Loss.

## Abstract

Ziyun Ointment, a traditional Chinese herbal remedy, is often applied externally; however, its physiological effects on the skin have not been thoroughly studied. This study aimed to assess the skin parameters of psoriasis patients before and after 12 weeks of Ziyun Ointment application. Methods: 30 participants were recruited and classified into mild, moderate, and severe. The pre-test and post-test measurements of skin parameters, including melanin and erythema index, stratum corneum lipids, stratum corneum hydration, and transepidermal water loss (TEWL), were conducted using the non-invasive Cutometer Dual MPA580. Result: 80% of the patients improved PASI, and the PASI score was significantly reduced by 2.58 ± 1.35 (*p* < 0.05). After continuous use of Ziyun Ointment for 49 days, the psoriasis area of all subjects was significantly reduced by 42% ± 4%, especially in the erythema index (*p* = 0.028). Ziyun Ointment significantly reduced the degree of skin transepidermal water loss by 26% (*p* = 0.04). Conclusion: Ziyun Ointment retains the hydration in the skin and reduces the loss of moisture from the skin. We also suggest that Ziyun Ointment be used for at least 12 weeks to observe significant improvements. Ziyun Ointment has a physiological significance in maintaining skin moisture.

## Introduction

Many diseases can cause skin pigmentation or leave skin spots of varying shades when they occur. The most common is skin discoloration caused by psoriasis, eczema, or acne 1. Cutaneous pigmentation is an important phenotypic trait, and the mechanism is that tyrosinase converts the essential amino acid tyrosine into dopamine and melanin. Then, the melanin is packaged in melanosomes and transferred to keratinocytes 2. Immoderate melanin synthesis can cause pigmentation disorders that are associated with psychosocial impacts. Skin pigmentation can cause distress to patients and even affect their self-confidence 3. One of the common reasons for seeking treatment is appearance, as skin pigmentation or inconsistent shades can cause stress and social stigma for patients. Therefore, how to reduce pigmentation is a very important dermatology issue.

About 0.51% to 11.43% of the world's adult population suffers from psoriasis 4. The characteristic dermatologic symptoms are thick, raised, and red patches with silver-white scales all over the body, especially on the scalp, elbows, and knees 5. Psoriasis is a chronic, autoimmune, inflammatory disorder 6-8. Psoriasis has a lot to do with Tumor necrosis factor-alpha, dendritic cells, T-cells, and leukocytes in regulating keratinocytes and melanocytes 9, 10. Depending on the situation of a psoriasis patient, there are different treatments. For mild psoriasis patients, topical medications such as corticosteroids, retinoids, Vitamin D analogs, etc., are suggested. For moderate to severe cases, doctors may apply biologics or phototherapy 11. However, corticosteroid 'phobia' is another chronic usage negative response showing cutaneous atrophy and even adrenal toxicities 12-15. Although symptoms are alleviated, varying shades of skin discoloration or spots may remain. Therefore, how to reduce psoriasis symptoms or pigmentation is still a topic of discussion.

Ziyun Ointment, a traditional Chinese medicine whose main active component, is shikonin 16. Ziyun Ointment has been widely used all over Asia to treat skin disorders such as macular eruption, carbuncles, measles, burns, and inflammation 17-19. Ziyun Ointment, the active principle in the root of Lithospermum erythrorhizon, has demonstrated its ability to the treatment of burns, anal ulcers, hemorrhoids, and skin wounds. Ziyun Ointment has considerable benefits, low toxicity, and is anti-inflammatory 20-22. Some report the hypopigmentation effect of Ziyun Ointment via inhibiting cellular tyrosinase activity, subsequently ameliorating melanin production 3, 23. Ziyun Ointment has also been studied in the treatment of psoriasis or eczema. Ziyun Ointment can reduce pigmentation by inhibiting tyrosinase activity, stimulated by α-MSH. This leads to decreased melanin production in B16F10 cells and the skin of C57BL/6J mice 3. Previous studies indicated that Ziyun Ointment inhibits IL-17-induced keratinocyte proliferation and the secretion of related inflammatory cytokines 24. Ziyun Ointment also inhibits HaCaT cell proliferation and induces cell apoptosis by regulating the p38 MAPK and JNK/MAPK signaling pathways 25. Ziyun Ointment inhibited the proliferation of colorectal cancer cells *in vitro*^26^. However, these studies were limited to *in vitro* cells, and there is a lack of follow-up studies on Ziyun Ointment application in skin patients. There is currently no literature exploring the effect of Ziyun Ointment on skin discoloration or spots in patients with psoriasis.

This study aimed to investigate the effects of Ziyun Ointment on skin physiological parameters (including melanin, erythema index, stratum corneum lipids, hydrates, and transepidermal water loss) in patients with psoriasis. The effects of treatment time on physiological parameters were further analyzed.

## Materials and Methods

### Reagents

Ziyun Ointment (commercial name: ITEPSC) was purchased from Prosche Biomed Co (Taiwan).

### Study population

All authors certify that the work described complies with the World Medical Association Declaration of Helsinki on experimental materials involving human subjects. This manuscript also complies with the Recommendations for the Conduct, Reporting, Editing, and Publication of Scholarly Work in Medical Journals and seeks to include representative human populations (sex, age, and ethnicity) by these recommendations. Informed consent was obtained for experiments conducted on human subjects/samples. This study was a randomized, controlled, single-center, preliminary, open-label trial. This study respects the privacy of human subjects and approval was obtained from the Institutional Review Board of the National Chung Shan Medical University Hospital, Taichung, Taiwan (CSMUH IRB number: CS2-18127). From December 2018 to December 2019, we recruited participants at the Department of Dermatology, Chung Shan Medical University Hospital, Taichung, Taiwan. A total of 32 patients diagnosed with chronic plaque-type psoriasis were enrolled in this study. Eligible participants were adults aged between 20 and 100 years, presenting with mild to moderate psoriasis, defined as a Psoriasis Area and Severity Index (PASI) score of less than 15. Patients were required to demonstrate the ability to apply the assigned topical medication three times daily—specifically in the morning upon waking, around dinner time, and at night before sleep—either independently or with assistance. Throughout the study period, participants were instructed not to initiate any new skin prescriptions or skincare regimens, refrain from consuming nutraceuticals or contraceptives that could influence skin condition, and avoid any aesthetic treatments. Additionally, sun exposure was to be minimized to less than 30 minutes per day. Exclusion criteria were carefully considered to minimize confounding factors. Patients were excluded if they had received aesthetic treatments within the month before enrollment or had used systemic corticosteroids or biologics in the preceding month.

While common complications of psoriasis, such as psoriatic arthritis, chronic liver disease, chronic kidney disease, metabolic syndrome, diabetes, and cardiovascular disease, were recorded but not taken as exclusion criteria. 32 patients were recruited in this trial, while two patients dropped out due to military careers in another city (1 patient) and loss to follow-up (1 patient). Therefore, a total of 30 completed the trial.

### Study Design

Ziyun Ointment would be batch-prescribed four times per day according to the size of the plaques, and the ointment was to be applied in a thin, even layer over the affected skin areas. The study period was 12 weeks, with 5 consultations at weeks 1, 3, 7, and 11 with the participants' dermatologists. Lithospermum erythrorhizon extracts ITEPSC (also called Tzu-Yun ointment were purchased from Prosche Biomed Co., Ltd., Taichung, Taiwan. Participants were asked to follow the instructions they were taught on how to apply Tzu-Yun ointment until the day before the measurement. Despite all lessons being thoroughly gone, the intervention would not be stopped until the whole course was completed. The treatment would be instantly stopped when any adverse effects, such as allergies.

### The Study Tools- Psoriasis Area Severity Index

Psoriasis severity was assessed by the psoriasis area and severity index (PASI) before inclusion in the study 27. PASI was a common assessment method for psoriasis, and dermatologists determined PASI scores. Based on four areas (head, arms, trunk, and legs) were assessed by the degree of erythema (redness), induration (thickness), and desquamation (scaling). This study used the Psoriasis Area Severity Index (PASI) as a reliable measurement^28^. Combining the scores consisted of PASI of psoriasis. Dermatologists evaluated patients' psoriasis symptoms covered area in four regions as follows: head and neck (H), upper limbs (UL), trunk (T) and lower limbs (LL), and gave a score on a visual analog scale of 1 (0-9%), 2 (10-29%), 3 (30-49%), 4 (50-69%), 5 (70-89%), or 6 (90-100%) according to lesion area percentage. The other essential factor in this scoring system is the severity of erythema (E), induration (I), and desquamation (D) grading on a scale of 0 to 4 as 0 (none), 1 (mild), 2 (moderate), 3 (severe), or 4 (very severe). During doctor consultations, every patient was taken photos for record purposes before and calculated by the formula below 29:

0.1 (E_H_ + I_H_ + D_H_) A_H_ + 0.2 (E_UL_ + I_UL_ + D_UL_) A_UL_ + 0.3 (E_T_ + I_T_ + D_T_) A_T_ + 0.4 (E_LL_ + I_LL_ + D_LL_) A_LL_

### Skin Parameters and Test Equipment

Skin parameters discussed in this study are melanin and erythema index, stratum corneum lipids, stratum corneum hydration, and TEWL. All measured with Cutometer® Dual MPA 580 (in arbitrary units; Courage and Khazaka Electronic GmbH, German) as previously manuscript 30. The measurements were taken at an average ambient room temperature of 25 ± 27°C. Measurements were taken both before and after applying Ziyun Ointment ointment. Stratum corneum lipids were taken once, while the other parameters were measured five times, using the averages for analysis.

### Statistical analysis

Clinical data will be analyzed using SPSS version 20.0. Between-group comparisons of normally distributed continuous variables will be performed using independent-sample t-tests, while within-group comparisons (pre- and post-treatment) will be conducted using paired-sample t-tests. For variables that do not meet normality assumptions, appropriate nonparametric tests (e.g., Mann-Whitney U or Wilcoxon signed-rank test) will be employed. Analysis of variance (ANOVA) will be used where applicable for comparing continuous variables across multiple groups. All statistical tests will be two-sided, and a *p*-value < 0.05 will be considered statistically significant.

## Results

### Distribution and characteristics of volunteers

**Figure 1:**
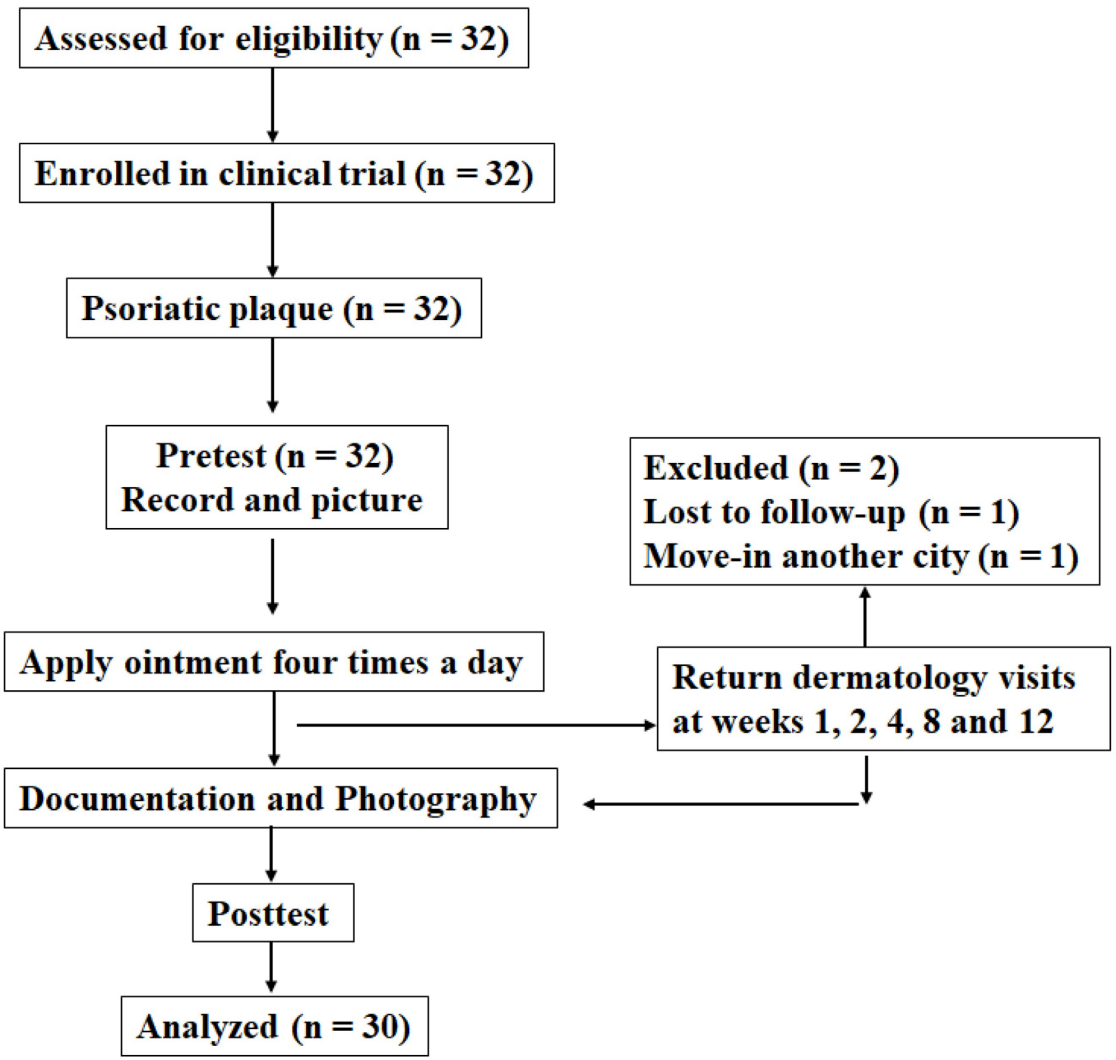
** Flow chart of the psoriasis clinical trial.** The dermatologist explains the research objectives, and the drug is applied and recorded with the consent of the subjects.

After detailed explanations and assessment for eligibility by dermatologists, 32 volunteers were willing to participate in the study. Common complications of psoriasis, such as psoriatic arthritis, chronic liver disease, chronic kidney disease, metabolic syndrome, diabetes, and cardiovascular disease, were recorded. Demographic characteristics, namely sex, age, psoriasis duration, PASI score, and complications, were recorded. This study included 10 (31.25%) men and 22 (68.75%) women. The age range of the total 32 participants was 49.8-25.6 years. The history of psoriasis is divided into four stages based on ten years, namely less than ten years (25 %), 10-20 years (43.75%), 20-30 years (12.5 %), and more than 30 years (18.75 %). In addition to suffering from psoriasis, volunteers also had other concurrent diseases, such as 16 psoriatic arthritis (50%), 21 chronic liver disease (65.63%), 7 chronic kidney disease (21.88%), 8 metabolic syndrome (25%), 10 diabetes (31.25%), and 7 cardiovascular disease (21.88%) **(Table 1)**. Dermatologists evaluate the patient's psoriasis area, spot distribution, and spot color to calculate the PASI score comprehensively. The PASI score mean of the total participants was 4.09 ± 3.64. Then, the volunteers were divided into three groups: mild (n = 24), moderate (n = 3), and severe (n = 2). First, 32 participants were recruited to apply the Ziyun Ointment for 12 weeks. Patients applied Ziyun Ointment daily for 12 weeks, during which time they had five medical consultations with a dermatologist (weeks 1, 3, 7, and 11). During this period, patients were not allowed to have new skin-related prescriptions. They had a pre-test and a post-test. Two volunteers withdrew from the study for any reason, leaving the total number of volunteers at 30. We show the flowchart of this study **(Fig.1)**.

### Ziyun Ointment decreased erythema and transepidermal water loss index in psoriasis

**Figure 2:**
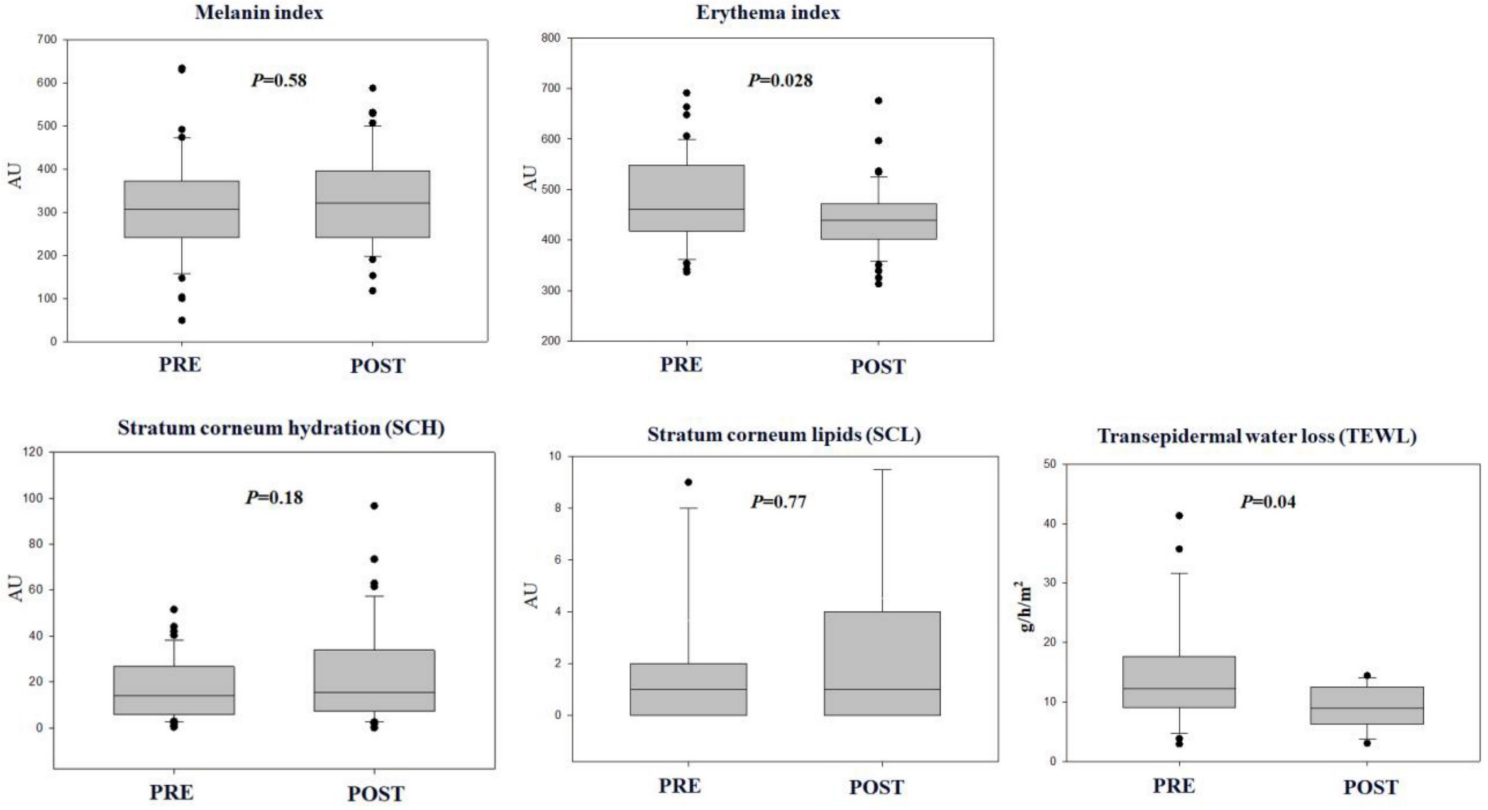
** Psoriatic patients' skin parameters.** Skin status was assessed using the Corneometer® CM580. Basic values for erythema and melanin were measured using a pigmentation probe (mexameter). Skin hydration and corneum lipid were assessed using a corneometer. Participants were recorded in the pretest and posttest. All values are presented as mean ± SD. All data were analyzed using a *t*-test. A *p*-value of less than 0.05 was considered to indicate statistical significance. *p < 0.05, **p < 0.01.

Skin barrier function parameters before and after applying Ziyun Ointment in psoriatic patients were compared. Using paired Student's T to test skin parameters: melanin and erythema index, stratum corneum lipids (SCL), and stratum corneum hydration (SCH) with Cutometer® Dual MPA580. The melanin index of psoriasis patients has not changed significantly before and after testing (318.27 ± 122.12 vs 331.99 ± 112.40 AU, *p* = 0.58). The erythema index was significantly lower post-testing psoriatic plaques (479.30 ± 88.72 vs 441.29 ± 67.93 AU, *p* = 0.028). The SCH index of psoriasis patients has not changed significantly before and after testing (18.04 ± 13.45 vs 23.13 ± 21.04 AU, *p* = 0.18). The SCL index of psoriasis patients has not changed significantly before and after testing (3.66 ± 11.64 vs 4.57 ± 14.55 AU, *p* = 0.77). TEWL was significantly lower post-testing psoriatic plaques (17.74 ± 9.23 vs 9.13 ± 3.58 AU, *p* = 0.04). Higher TEWL is often associated with skin barrier impairments **(Fig. 2)**.

Based on the results of the above skin parameters, the Ziyun Ointment can significantly reduce the erythema index and reduce the TEWL index, allowing the skin to retain water. Reduce patients' skin dryness and discomfort.

### Ziyun Ointment significantly improves mild psoriasis

**Figure 3:**
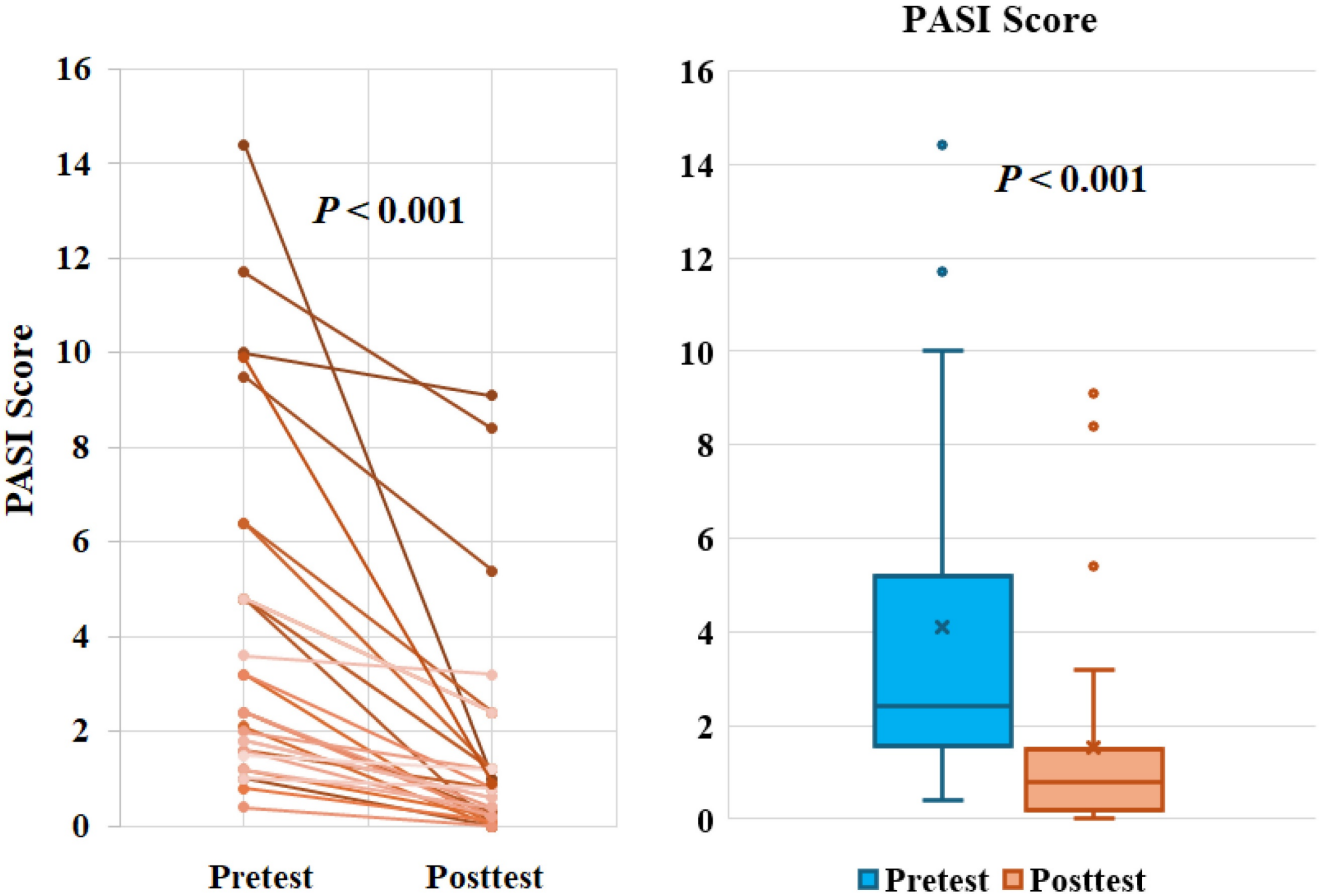
** Recording of PASI index. (A)** Skin PASI changes of 30 volunteers. The index changes between the values ​​before volunteers applied Ziyun Ointment and the 12 weeks of use. **(B)** Distribution of PASI. *p < 0.05, **p < 0.01.

**Figure 4:**
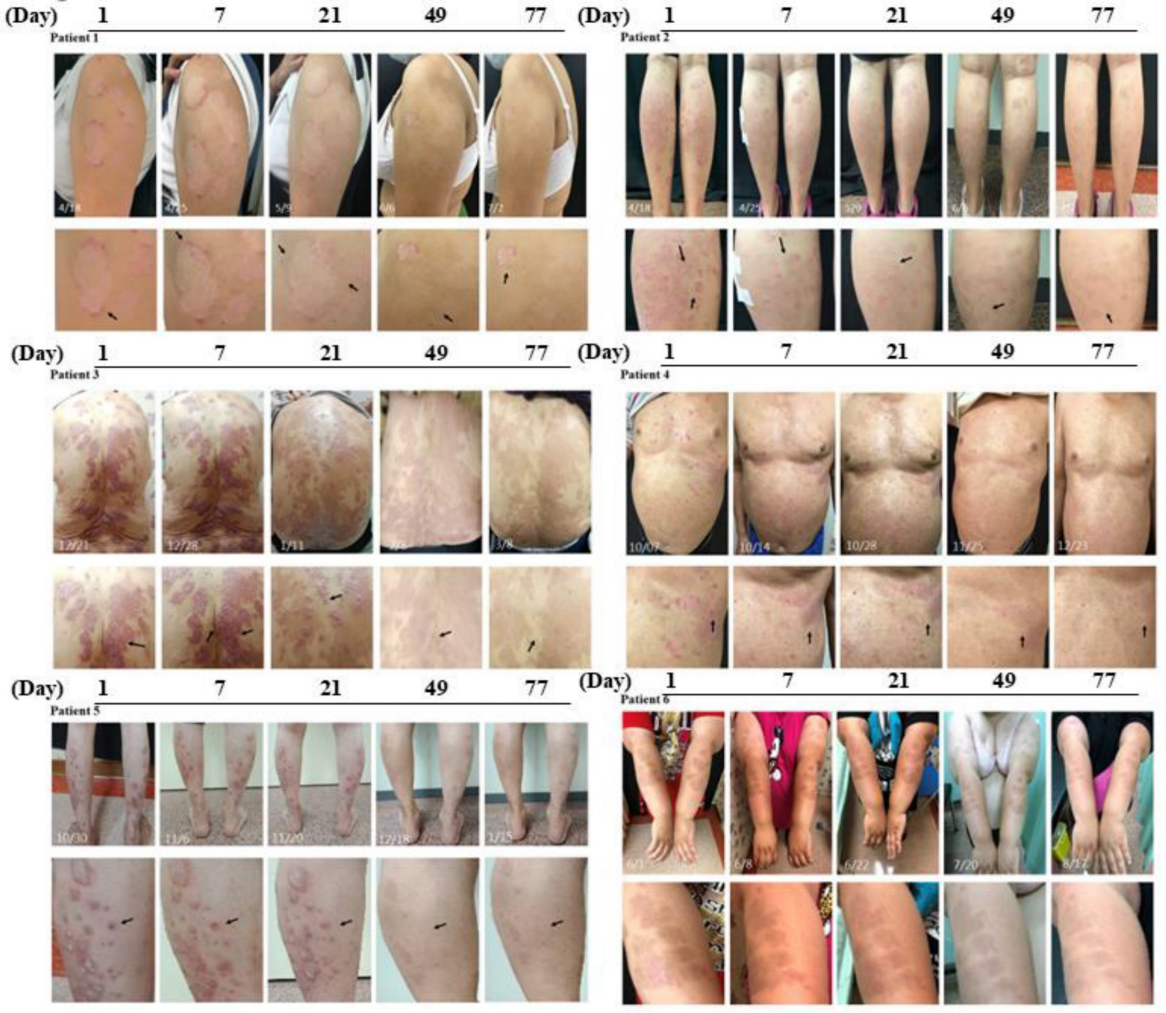
** Clinical cases presenting psoriasis in different locations. Several psoriasis patients were randomly selected, and clinical lesions in different parts were presented.** Patient** 1** has a large erythema on his left upper arm. The picture below shows a local dermoscope magnified 5 times. Black arrows indicate significantly improved skin areas. Patient **2** had diffuse psoriasis on both lower legs. Patient** 3** had significantly large areas of psoriasis on the back. Patient **4** had punctate psoriasis and protrusions on his chest. Patient **5** had obvious scaling and raised psoriasis on the left calf. Patient 6 had large areas of spots on the outer sides of both arms.

**Figure 5:**
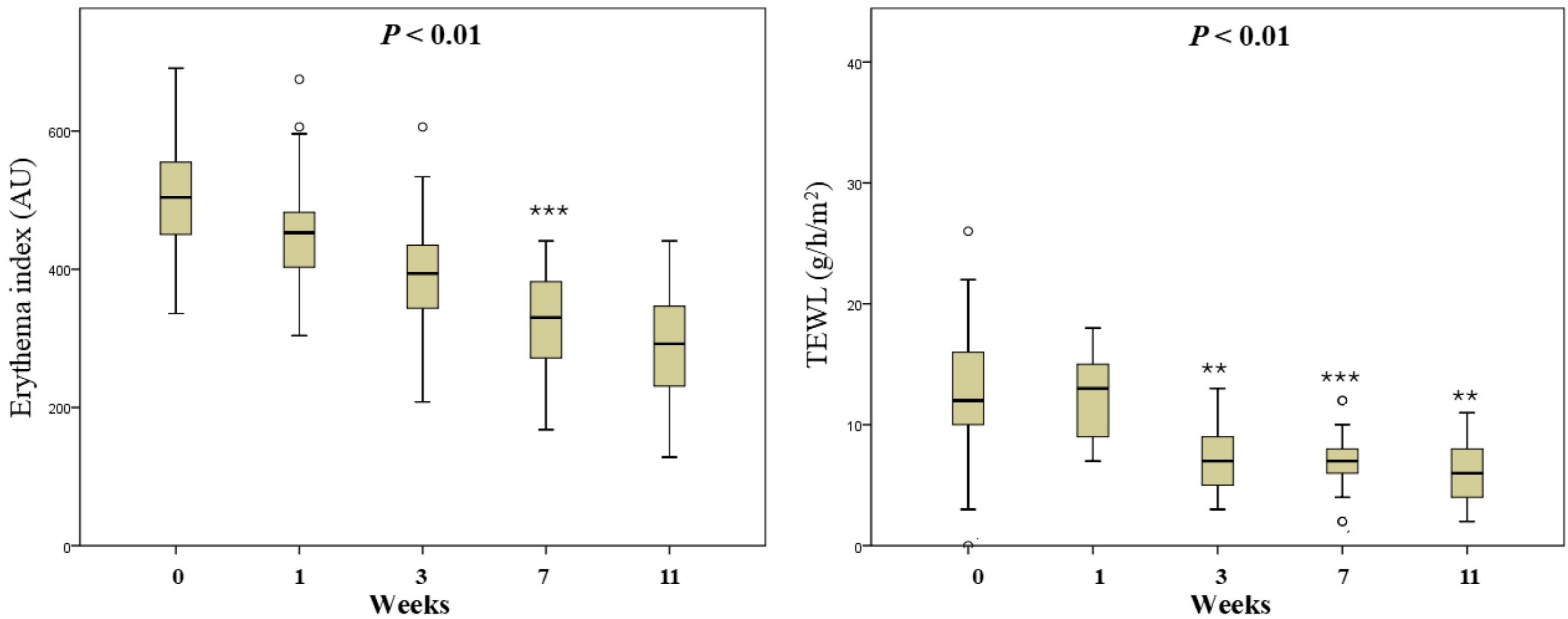
** Relationship between the application time of Ziyun Ointment and skin physiological parameters.** All subjects applied Ziyun Ointment to the affected area four times a day. Dermatologists observed the changes in the psoriasis area and took photos at fixed points in weeks 1, 3, 7, and 11. ANOVA was used to analyze the relationship between time of week and erythema and TEWL parameter values. (*p* < 0.05 is statistically significant, *** represents *p* < 0.001)

To explore the degree of improvement of Ziyun Ointment on psoriasis patients, they had their PASI scores evaluated by the dermatologist and were taken photos for record purposes at the beginning and the end. The PASI distribution determined by dermatologists was recorded. The magnitude of improvement with Ziyun Ointment was shown on the left **(Fig. 3)**. The mean of PASI of all subjects was 4.09 ± 3.64 before the test, the median was 2.4. After following the dermatologist's instructions and applying the medication for 12 weeks, the mean of PASI dropped to 1.51 ± 2.29, and the median was 0.8 (*p* < 0.001). All subjects experienced significant improvement in their psoriasis. Further, subjects were divided into three groups according to PASI. We classified participants into mild (PASI score less than or equal to 5), moderate (greater than or equal to 5 and less than 10), and severe (greater than or equal to 10). The proportion of patients achieving 75%, 90%, and 100% improvements in PASI score at 12-week follow-up (PASI 75, PASI 90, and PASI 100). In **Table 2**, fifty-four percent of patients achieved PASI 75 (> 75% improvement from baseline PASI) at 12 weeks, 25% achieved PASI 90, 21% achieved PASI 100. Subjects in the mild group showed significant improvement before and after use, regardless of whether they reached PASI 75 (*p* = 0.0001), PASI 90 (*p* = 0.016) or PASI 100 (*p* = 0.043). Eighty percent of all patients improved PASI response after three months of treatment. There was significant improvement, especially in the mild group. However, the number of people in the moderate and severe groups was small, so the improvement was not statistically significant. The patient's skin appearance and state from before applying Ziyun Ointment to three months after treatment was shown in **Fig. 4.** The patient's follow-up appointment was assessed by a dermatologist, and photos were taken. Psoriasis was diagnosed by the presence of white, characteristic plaques consisting of sharply bordered salmon-colored macules covered with silvery scales. The legs, arms, back, and chest are commonly affected. As the time of applying Ziyun Ointment increases, by the end of the 12-week course of treatment, the psoriasis lesions have been significantly improved (the black arrow), and the deposition of pigmented plaques has been reduced. Further observations through ANOVA statistical analysis showed that in the 7th week of using Ziyun Ointment, the erythema precipitation of psoriasis patients decreased, and the degree of epidermal water loss decreased (*p* < 0.001), which showed a significant effect (**Fig. 5**). “In participants' comorbidities and quality of life, all 30 participants reported that their comorbidities, including psoriatic arthritis, chronic liver disease, or other inflammatory conditions, were well-controlled during the study period, as confirmed during routine follow-up visits. Furthermore, 15 participants completed the Dermatology Life Quality Index (DLQI) questionnaire Supplementary Table 1, with all reporting total scores below 5, suggesting a minimal impact on quality of life and an overall improvement in self-perceived well-being after treatment.”

All of those results, the total study duration was 12 weeks, but significant improvements were first noted at week 7.

## Discussion

This study provides evidence that in psoriasis patient skin models, Ziyun Ointment can significantly reduce erythema in the skin physiological index and reduce the skin moisture loss index in psoriasis patients. This study confirms that Ziyun Ointment has the effect of maintaining skin moisture and whitening spots for patients with psoriasis. A human trial on 30 subjects confirmed that applying Ziyun Ointment for 45 days would significantly improve the condition, especially in patients with mild PASI.

Psoriasis is diagnosed based on characteristic plaques, such as areas of varying shades of red with irregular edges and the skin covered with silvery scales, persistent itching, and dryness. Most will be distributed on the patient's knees, elbows, scalp, and back. Dermatologists use the Psoriasis Area and Severity Index (PASI) constructed by Fredriksson and Pettersson to determine clinical severity 31. Psoriasis is easily affected by the environment, psychological factors, stress, diet, climate, etc., and the severity of the disease varies. The appearance of spots or scaling on the skin can cause low self-esteem and even depression. The empirical effects of Ziyun Ointment are clear from clinically confirmed results in numerous patients (**Fig.2,3,4**). Ziyun Ointment has a significant effect on reducing the exacerbation of patients with mild psoriasis (**Table 2**).

Some literature indicates that Ziyun Ointment, combined with other drugs, has the effect of treating psoriasis or skin diseases. Ziyun Ointment combined with methotrexate can regulate the polarization of macrophages both in imiquimod-induced psoriasis mice and LPS-stimulated RAW264.7 cells to constrain psoriasis progression, such as keratinocyte proliferation and angiogenesis 22, 32. However, there is no literature on Ziyun Ointment to prevent the pigmented and relative epidermal water loss spots caused by psoriasis. Ziyun Ointment can reduce inflammatory infiltration 23, 32-34, increase collagen deposition, and enhance angiogenesis in the diabetic rat model 35. Studies also indicated a Ziyun Ointment reduction in ICAM, KI67, and IL17 inflammation cytokine expression levels through the HIF-1 signaling pathway in HaCaT cells 33. Previous studies have shown that Ziyun Ointment remodels deep wound healin 35, 36. Ziyun Ointment may promote wound healing by reorganizing collagen and increasing water retention. However, these are cell experiments or animal tests, and cannot directly prove that shikonin or skin medications containing shikonin are effective for psoriasis. There is also a lack of literature to observe and verify this in most psoriasis patients. Psoriatic skin most commonly suffers from poor water retention. Therefore, if the water retention can be improved or the loss of epidermal moisture can be reduced, the speed of wound healing may be increased.

Psoriasis is a common immune-mediated inflammatory skin disease 37, 38. In clinical symptomatology, dry and scaly skin is caused by abnormal proliferation of epidermal keratinocytes in psoriasis 39. Scars or uneven skin spots can cause a variety of stresses on a person's appearance, both psychologically and physically 40. Psoriasis has a significant correlation with psychosocial status or even psychiatric disorders like anxiety, depression, and sleep deprivation, which may lead to a higher risk of cardiometabolic diseases and severe dermatologic conditions 41, 42. In sum, the bidirectional relationship between a patient's physical condition and psychiatric status is profound 43, 44.

Among other skin physiological parameters, Ziyun Ointment also promotes sebaceous glands (SG), compared to a median increase after use (although it does not reach statistical significance). Sebaceous glands are important skin appendages that lubricate the skin by secreting lipids, forming a protective barrier on the skin surface. Here, we have demonstrated Ziyun Ointment increases lipid secretion in psoriatic skin to protect the skin and reduce friction and dryness (**Fig.2**). We must also consider sun exposure, and our skin is the first barrier. Photo exposure (UV) was also one of the causes of worsening psoriasis 45. UVB can cause inflammation, dry skin, or erythema. Studies have indicated Ziyun Ointment mixed with beta-cyclodextrin complex could significantly increase the superoxide dismutase (SOD) activity in the skin 46.

Psoriasis is a difficult-to-treat and highly variable disorder that poses significant challenges for affected individuals. Most patients with psoriasis have other comorbidities, but these subjects were not excluded from this study. This study was mainly aimed at alleviating the discomfort and reducing the formation of erythema in patients with psoriasis, to improve the subjects' quality of life and self-confidence. While it is not life-threatening, it profoundly impacts patients' quality of life. The condition is characterized by large, discolored patches of skin, persistent itching, and dryness, causing constant discomfort. Therefore, identifying gentle, natural, and safe adjunctive therapies is paramount to alleviating symptoms and improving patients' well-being.

This study has several limitations that warrant attention. First, the sample size was relatively small, which may affect the generalizability of the findings. Second, the lack of long-term follow-up makes it difficult to evaluate the recurrence of psoriasis symptoms and the sustained efficacy of Ziyun ointment. Future studies with larger cohorts and extended follow-up periods are needed to address these limitations, enabling a more comprehensive interpretation of the results and offering valuable guidance for the development of effective clinical treatment strategies.

## Conclusion

Ziyun Ointment, a traditional Chinese herbal remedy, has been historically used for external skin application, yet its physiological effects have not been thoroughly investigated. In this study, we evaluated the impact of 12 weeks of topical Ziyun Ointment on skin barrier function in psoriasis patients. Significant clinical improvements were observed, with reductions in PASI scores, erythema index, and TEWL index. These findings suggest that Ziyun Ointment enhances skin hydration and integrity, offering a supportive role in psoriasis management.

## Supplementary Material

Supplementary table.

## Figures and Tables

**Table 1:** Patient Demographic and Clinical Characteristics.

	Psoriasis patient (N = 32)	
	N	%
Characteristics	Mean ± SD	Median (range)
Gender		
Male	22	68.75
Female	10	31.25
Age (year)	49.84 ± 155.64	53.50 (22-79)
Psoriasis Duration		
< 10 years	8	25.00
10-20 years	14	43.75
20-30 years	4	12.50
>30 years	6	18.75
PASI Score	4.09 ± 3.64	2.4 (0.4-14.4)
Complication		
Psoriatic arthritis	16	50.00
Chronic liver disease	21	65.63
Metabolic syndrome	8	25.00
Diabetes	10	31.25
Cardiovascular disease	7	21.88

**Table 2:** Ziyun Ointment of 12-week active therapy psoriasis: results on PASI responses.

Classified	PASI 75			PASI 90			PASI 100		
	N	%	*P*	N	%	*P*	N	%	*P*
Mild	13	54	0.0001**	6	25	0.016**	5	21	0.043**
Moderate	2	50	0.16	1	25	N/A	0		
Severed	2	33	N/A	1	33	N/A	0		

PASI, Psoriasis Area and Severity Index. ** The proportion of patients achieving 75%, 90%, and 100% improvements in PASI score at 12-week follow-up (PASI 75, PASI 90, and PASI 100).
