# Supplementary material for: Clinical Efficacy of Topical Ziyun Ointment in Improving Skin Hydration and Reducing Transepidermal Water Loss in Psoriasis
Source: Int J Med Sci. 2025 Sep 12;22(15):4026–35. doi: 10.7150/ijms.118035 (PMC12492351; doi:10.7150/ijms.118035)
Supplement: Supplementary file 1 — Supplementary table. [file ijmsv22p4026s1.pdf]

Supplementary Table 1.

Table 1. The DLQI questionnaire

Headings	Questions	Answers	Score
**1. Symptoms and feelings**	Q1: Over the last week, how itchy, sore, painful, or stinging has your skin been?	Very much<br>A lot<br>A little<br>Not at all	3<br>2<br>1<br>0
	Q2: Over the last week, how embarrassed or self-conscious have you been because of your skin	Very much<br>A lot<br>A little<br>Not at all	3<br>2<br>1<br>0
**2. Daily activities**	Q3: Over the last week, how much has your skin interfered with you going shopping or looking after your home, or garden	Very much<br>A lot<br>A little<br>Not at all<br>Not relevant	3<br>2<br>1<br>0<br>0
	Q4: Over the last week, how much has your skin influenced the clothes you wear?	Very much<br>A lot<br>A little<br>Not at all<br>Not relevant	3<br>2<br>1<br>0<br>0
**3. Leisure**	Q5: Over the last week, how much has your skin affected any social or leisure activities?	Very much<br>A lot<br>A little<br>Not at all<br>Not relevant	3<br>2<br>1<br>0<br>0
	Q6: Over the last week, how much has your skin made it difficult for you to do any sport?	Very much<br>A lot<br>A little<br>Not at all<br>Not relevant	3<br>2<br>1<br>0<br>0
**4. Work and school**	Q7: Over the last week, has your skin prevented you from working or studying?	Yes<br>Not	3<br>0
	If 'no', over the last week, how much has your skin been a problem at work and studying?	A lot<br>A little<br>Not at all	2<br>1<br>0
**5. Personal relationships**	Q8: Over the last week, how much has your skin created problems with your partner or any close friends or relatives?	Very much<br>A lot<br>A little<br>Not at all<br>Not relevant	3<br>2<br>1<br>0<br>0
	Q9: Over the last week, how much	Very	3

	has your skin caused any sexual difficulties?	much<br>A lot<br>A little<br>Not at all<br>Not<br>relevant	2<br>1<br>0<br>0
**6. Treatment**	Q10: Over the last week, how much of a problem has the treatment for your skin been, for example, by making your home messy, or by taking up time?	Very<br>much<br>A lot<br>A little<br>Not at all<br>Not<br>relevant	3<br>2<br>1<br>0<br>0

Each question was scored on a scale from 0 to 3 (0 = not at all, 1 = a little, 2 = a lot, 3 = very much), and the scores for all ten questions were summed to obtain the total DLQI score, which ranged from 0 to 30 (0–1 = no effect on the patient’s quality of life, 2–5 = small effect, 6–10 = moderate effect, 11–20 = very large effect, 21–30 = extremely large effect).
